# Prevalence, Predictors, and Characteristics of Waterpipe Smoking Among Jazan University Students in Saudi Arabia: A Cross-Sectional Study

**DOI:** 10.5334/aogh.2912

**Published:** 2020-07-29

**Authors:** Sarah Salih, Samy Shaban, Zainab Athwani, Faizah Alyahyawi, Sana Alharbi, Fatima Ageeli, Arwa Hakami, Atheer Ageeli, Ohoud Jubran, Saleha Sahloli

**Affiliations:** 1 Jazan University, SA

## Abstract

**Background:**

Waterpipe smoking (WPS), also called shisha, has considerable short and long-term effects on human health. WPS has become increasingly popular among the youth in Jazan society. Hence, this study is aimed to assess the prevalence, predictors, and characteristics (knowledge and attitude) of WPS among male and female students at Jazan University.

**Methods and materials:**

A descriptive, cross-sectional study was conducted among 405 students. Data were collected using a self-administered validated questionnaire. The prevalence and predictors of WPS and the association between important socio-demographic factors (sex, type of college, parents smoking, home mate smoking and close friend smoking) were studied.

**Results:**

The prevalence of WPS among students was high, approximately 34.0%. The prevalence rate was significantly higher in males (42.5%, n = 74) than in females (27.0%, n = 57) (p = 0.001). The main predictors of WPS were: being male (OR = 1.99, 95% CI [1.30, 3.06], p = 0.001), believing that shisha smoking is less harmful & addictive than cigarettes (OR = 3.84, 95% CI [1.88, 7.83], p < 0.001 and 3.80, 95% CI [2.0, 7.11], p < 0.001, respectively), and having a close friend who smokes (OR = 6.85, 95% CI [3.84, 12.22], p < 0.001).

**Conclusions:**

WPS prevalence among Jazan University students was high, and the most influential factors were being male, having smoker housemates and friends, and having incorrect thoughts and beliefs.

## 1. Introduction

Waterpipe smoking (WPS), which is otherwise called shisha, narghile, and Gozaor hookah, has been one of the most common strategies for tobacco use in developing nations for approximately 400 years [1,2,3,4]. Its device consists of a head, body, water bowl, and hose [5].

WPS contains tobacco-specific nitrosamines and glycerol nicotine, which are derived from raw materials, and it produces chemical substances (such as carbon monoxide (CO)), which are synthesised during smoking, and produces 34 polyaromatic hydrocarbons, which are synthesised and transmitted when smoking [6].

WPS has been used for around 400 years [1]; the Arabian Peninsula, Turkey, India and Pakistan are among the countries where WPS has become increasingly popular [7].

WPS has considerable effects, both short and long term, on human health. Its short-term health effects include headache, nausea, lethargy, and fainting. Waterpipe smoking also impairs baroreflex control, which helps control blood pressure. Various long-term health effects may be caused including pulmonary diseases (e.g. chronic obstructive pulmonary disease) and coronary heart disease. WPS appears to increase the risk of several cancers such as lung, oral, oesophageal, and gastric cancer [3,8,9]. WPS also leads to numerous communicable diseases and respiratory diseases such as influenza, hepatitis and TB [10,11].

Mahfouz et al. studied the prevalence of tobacco use and its associated factors in 4100 students, both male and female, at Jazan University, and states that (according to the World Health Organization) around four million people die annually due to tobacco use worldwide [12]. The percentage of males who used WPS was 12.1% (95% Confidence Interval [CI]: 10.6–13.8), whereas that of females was 2.5% (95% CI: 1.8–3.4). Hassan et al. conducted a study in Riyadh, Saudi Arabia in 2014, with 156 students from Al-Ghad International College, to assess the prevalence of tobacco smoking [13]. The study showed of those surveyed: 42.3% were current smokers, 17.9% were past smokers, 34.8% were cigarette smokers and 21.2% were WPS users. In 2010, Taha et al. conducted a cross-sectional study at Lord Faisal College in Dammam City, Saudi Arabia, to investigate the prevalence of WPS among male understudies from three medical colleges [14]. The general prevalence of WPS was found to be 12.6% (n = 47).

The worldwide prevalence of WPS has also been studied. In Aleppo, Syria (2014), WPS prevalence was 25.5% and 4.9% among male and female university students, respectively [15]. In Karachi, Pakistan (2009), the prevalence among 422 college students was 45.2% [1]. In the United Kingdom a 2015 study was done to measure the prevalence and patterns of water-pipe tobacco use among 2217 student from six universities, it found a total of 66.0% (n = 1409) reported having ever tried water-pipe tobacco, and 14.4% (n = 300) reported past-30 day water-pipe tobacco use [16].

Numerous people wrongly believe that WPS is less harmful than cigarette smoking [2,3,9]. Also, the research team believes that WPS is gaining popularity among younger generations, among females and in social gatherings. Waterpipe smoking is being offered in many restaurants and coffee shops in the Jazan region. Therefore, this study focuses on the prevalence of WPS and its predictors among Jazan University students which were not assessed by previous studies.

## 2. Materials and methods

### 2.1. Study design, settings, and population

This study is an observational, descriptive, cross-sectional, and web-based survey to assess the prevalence of WPS and its determinants among male and female students in Jazan University. The inclusion criteria were students at Jazan University enrolled in the academic year 2018. Excluded from the study were visiting students from outside Jazan University.

### 2.2. Sample size and sampling procedures

Using a multistage stratified simple random sampling, a study sample from Jazan University students was selected. First, the colleges were divided into two strata (medical and nonmedical) and then the colleges were randomly selected by lottery method from each stratum according to their number. Three colleges were selected from the medical stratum (Medicine, Applied Medical Science and Pharmacy) and another three colleges were selected from the nonmedical stratum (Science, Computer Sciences and Information Systems and Education).

The calculated sample size was divided between these six colleges proportionate to the size of students in these programs. Participating students within these colleges were selected by simple random technique, and they received a web-based survey via social networks. They used their own internet to fill out the questionnaire and at a time and place of their convenience.

The calculated sample size for the study was 405 participants, and it was calculated by the following equation for a cross-sectional study: *n* = *z*^2^ × (p) × (1p – p)/d^2^, where the 95% CI, the anticipated population proportion (p) of the sample, was estimated to be 50%. This team felt 50% was the safest choice for p because the required sample size is largest when p = 50% and when the required absolute precision on either side of the anticipated population proportion (in percentage points) is d = 5% (0.05). An additional 5% was allotted for the anticipated non-response rate.

### 2.3. Data collection and quality control

Data were collected using a 45-item self-administered questionnaire, which had been used in previous research on the same population [17], and we obtained the necessary permission to use it. The questionnaire was divided into three parts. The first part contains five-item demographic and background data. The second part contains 11 items on respondents’ knowledge and attitude regarding WPS, its effect on health, and knowledge about tobacco and nicotine (which are present in WPS). The third part, which includes 29 items, pertains to the practice of frequent WPS and the reasons that lead the participant to use it. The questionnaire was revised by some experts in the epidemiology and behavioral and social science departments to evaluate the validity of its content according to a previous study by Bahri et al. [17] In addition, face validity was assessed by piloting the questionnaire on a sample of 30 students to ensure clarity and to guarantee that all participants would be able to answer the questionnaire completely.

### 2.4. Data management and analysis

The collected data were verified manually, encoded to a personal computer, and then analysed using the Statistical Package for the Social Sciences version 22. Descriptive statistics were calculated for study variables – e.g., frequency and percentage for qualitative variables, and mean and standard deviation for quantitative variables. Appropriate significance tests (such as chi-square) were applied. Binary logistic regression analysis was performed to detect the predictors of WPS; p < 0.05 was considered to be statistically significant.

### 2.5. Ethical consideration

Ethical approval was obtained from the Institutional Review Board of Jazan University, Kingdom of Saudi Arabia. Data privacy and confidentiality were maintained throughout the project. Furthermore, informed consent was a prerequisite to complete the online survey.

## 3. Results

### 3.1. Socio-demographic data of included participants

A total of 385 students responded to the questionnaire (response rate 95%). The mean participant age was 22.03 years (Table 1). Most participants (54.8%) were female, with males constituting only 45.2%. The majority of students (94, 24.4%) were from the Faculty of Applied Medical Sciences followed by the Faculty of Education (87, 22.6%) and the Faculty of Medicine (74, 19.2%). Regarding residence, more students (50.9%) were from rural areas than from urban areas (49.1%). Moreover, 74 (19.2%) students had one or both of their parents who smoked, 137 (35.5%) students had a housemate who smoked and 245 (63.6%) students had a close friend who smoked.

**Table 1:** Socio-demographic data.

Socio-demographic		Male	Female	Total	P-value

**Age, years**		20.84 ± 1.31	22.17 ± 1.29	21.38 ± 1.45	**<0.001**
Study	Medical	109 (62.6%)	84 (39.8%)	193 (50.1%)	**<0.001**
	Non-medical	65 (37.4%)	127 (60.2%)	192 (49.9%)	
Faculty (n, %)	Faculty of Applied Medical Sciences	50 (28.7%)	44 (20.9%)	94 (24.4%)	**<0.001**
	Faculty of Pharmacy	14 (8%)	11 (5.2%)	25 (6.5%)	
	Faculty of Science	28 (16.1%)	42 (19.9%)	70 (18.2%)	
	Faculty of Medicine	45 (25.9%)	29 (13.7%)	74 (19.2%)	
	Faculty of Computer Sciences and Information Systems	23 (13.2%)	12 (5.7%)	35 (9.1%)	
	Faculty of Education	14 (8%)	73 (34.6%)	87 (22.6%)	
Residency	Rural	95 (54.6%)	101 (47.9%)	196 (50.9%)	0.219
	Urban	79 (45.4%)	110 (52.1%)	189 (49.1%)	

### 3.2. Prevalence of WPS

The prevalence of WPS among all students at Jazan University was 34.0%. The prevalence rate was significantly higher in males (42.5%, χ^2^ = 10.225, p = 0.001) than in females (27.0%, χ^2^ = 10.225, p = 0.001). Regarding the type of college, the prevalence rate of medical colleges (31.1%) and of nonmedical colleges (37.0%) was insignificant (χ^2^ = 1.488, p = 0.223). The prevalence rate among urban and rural residence was also insignificant (χ^2^ = 1.305, p = 0.253). Social environment characteristics show that parents who smoke were insignificantly associated with frequent WPS in students (37.8%) (χ^2^ = 0.593, p = 0.441). Furthermore, the prevalence of WPS among the housemates of students was significantly high (48.2%, χ^2^ = 18.968, p < 0.001). A proportion of students who had the habit of smoking with close friends was found to be significant (46.9%, χ^2^ = 50.044, p < 0.001) (Table 2).

**Table 2:** Frequent water-pipe smoking and Socio-demographic factors.

Socio-demographic factors	Frequent Smoking				*χ^2^	P-Value

	Yes		No			

	N	%	N	%		

**Student Gender**						
Male	74	42.5	100	57.5		
Female	57	27.0	154	73.0	10.225	**0.001**
Total	131	34.0	254	66.0		
**Type of College**						
Medical	60	31.1	133	68.9		
Nonmedical	71	37.0	121	63.0	1.488	**0.223**
Total	131	34.0	254	66.0		
**Residence**						
Urban	59	31.2	130	68.8		
Rural	72	36.7	124	63.3	1.305	**0.253**
Total	131	34.0	254	66.0		
**Parent Smoking**						
Yes	28	37.8	46	62.2		
No	103	33.1	208	66.9	0.593	**0.441**
Total	131	34.0	254	66.0		
**Home-Mate Smoking**						
Yes	66	48.2	71	51.8		
No	65	26.2	183	73.8	18.968	**<0.001**
Total	131	34.0	254	66.0		
**Close-Friend Smoking**						
Yes	115	46.9	130	53.1		
No	16	11.4	124	88.6	50.044	**<0.001**
Total	131	34.0	254	66.0		

* χ^2^, chi-square.

### 3.3. Knowledge and attitude towards WPS

Regarding attitude, 6.2% of participants smoke shisha 1-3 times weekly, and 2.3% smoke 4-6 times weekly. The most preferred place for smoking is with gatherings of friends (13.8%). Only 5.7% think that smoking shisha makes them look good. About 24% of participants smoke for experience and curiosity, 11.7% because of friends, and 9.4% due to boredom and emptiness. Five percent of the participants smoke cigarettes as well as smoking shisha. 7% of respondents chew qat while smoking shisha (Table 3).

**Table 3:** Knowledge and attitude of included participants towards WPS.

Knowledge parameters		Male	Female	N (%)	P-value*

Does the water pipe smoking cause damage on health?	Agree	160 (92%)	205 (97.2%)	365 (94.8%)	**0.032**
	Disagree	7 (4%)	1 (0.5%)	8 (2.1%)	
	I do not Know	7 (4%)	5 (2.4%)	12 (3.1%)	
Does the shisha contain nicotine as cigarette?	Agree	130 (74.7%)	136 (64.5%)	266 (69.1%)	**0.039**
	Disagree	8 (4.6%)	8 (3.8%)	16 (4.2%)	
	I do not Know	35 (20.1%)	67 (31.8%)	102 (26.5%)	
Does the shisha contain tobacco as cigarette?	Agree	114 (65.5%)	112 (53.1%)	226 (58.7%)	**0.003**
	Disagree	21 (12.1%)	17 (8.1%)	38 (9.9%)	
	I do not Know	38 (21.8%)	79 (37.4%)	117 (30.4%)	
Does the using of shisha between more than one person can conveydiseases for you?	Agree	145 (83.3%)	185 (87.7%)	330 (85.7%)	0.119
	Disagree	7 (4%)	11 (5.2%)	18 (4.7%)	
	I do not Know	21 (12.1%)	13 (6.2%)	34 (8.8%)	
Do you think smoking shisha will be less addictive than cigarettes smoking?	Yes	76 (43.7%)	78 (37%)	154 (40%)	0.336
	No	60 (34.5%)	80 (37.9%)	140 (36.4%)	
	I do not Know	36 (20.7%)	53 (25.1%)	89 (23.1%)	
Do you think smoking shisha is less harmful than cigarettes?	Yes	39 (22.4%)	38 (18%)	77 (20%)	0.290
	No	101 (58%)	139 (65.9%)	240 (62.3%)	
	I do not Know	33 (19%)	33 (15.6%)	66 (17.1%)	
Do you think the filter mechanism that in shisha make it less harmful?	Yes	35 (20.1%)	21 (10%)	56 (14.5%)	**0.013**
	No	71 (40.8%)	106 (50.2%)	177 (46%)	
	I do not Know	67 (38.5%)	83 (39.3%)	150 (39%)	
Do you think smoking shisha in different flavor like apple flavor makes it less harmful than flavorless one?	Yes	28 (16.1%)	22 (10.4%)	50 (13%)	0.217
	No	92 (52.9%)	115 (54.5%)	207 (53.8%)	
	I do not Know	52 (29.9%)	73 (34.6%)	125 (32.5%)	
**Attitude Parameters**					

Have you ever try to smoke shisha?	Yes	74 (19.2%)	57 (14.8%)	131 (34%)	**0.001**
	No	100 (26%)	154 (40%)	254 (66%)	
How many times you smoke shisha in a week?	Not weekly	41 (11%)	44 (11.8%)	85 (22.1%)	**0.001**
	1–3 times	17 (4.6%)	7 (1.9%)	24 (6.2%)	
	4–6 times	7 (1.9%)	2 (0.5%)	9 (2.3%)	
	More than 6 times	7 (1.9%)	2 (0.5%)	9 (2.3%)	
Where do you prefer smoking shisha?	At home	9 (2.4%)	14 (3.8%)	23 (6%)	**0.001**
	At a coffee shop	30 (8.1%)	11 (3%)	41 (10.6%)	
	Private gatherings with friends	28 (7.6%)	25 (6.8%)	53 (13.8%)	
	Other	4 (1.1%)	13 (3.55%)	17 (4.4%)	
Do you think that you will smoke some day?	Yes	51 (29.3%)	30 (14.2%)	81 (21%)	**0.001**
	No	99 (56.9%)	147 (69.7%)	246 (63.9%)	
	I do not know	24 (13.8%)	34 (16.1%)	58 (15.1%)	
Do you think smoking shisha makes you look good?	Yes	9 (5.2%)	13 (6.2%)	22 (5.7%)	0.126
	No	155 (89.1%)	194 (91.9%)	349 (90.7%)	
	I do not know	10 (5.7%)	4 (1.9%)	14 (3.6%)	
What is the cause that made you smoke the shisha for the first time?	Experience and curiosity	41 (30.6%)	49 (36.6%)	90 (23.4%)	**<0.001**
	Friends	30 (22.4%)	15 (11.2%)	45 (11.7%)	**0.005**
	Family	3 (2.2%)	2 (1.5%)	5 (1.3%)	0.051
	Boredom and emptiness	25 (18.7%)	11 (8.2)	36 (9.4%)	**0.003**
	Other	0	2 (1.5%)	2 (5%)	**0.015**
Do you Hide the fact that you smoke shisha from?	Parents	41 (31.3%)	23 (17.6%)	64 (16.6%)	**0.003**
	Brothers	19 (14.5%)	14 (10.7%)	33 (8.6%)	**0.024**
	Friends	1 (0.8%)	3 (2.3%)	4 (1%)	**0.016**
	All of them	7 (5.3%)	13 (9.9%)	20 (5.2%)	**0.009**
	No one	25 (19.1%)	18 (13.7%)	43 (11.2%)	**0.003**
Do you smoke cigarettes besides smoking the shisha?	Yes	18 (4.7%)	3 (0.8%)	21 (5.5%)	**<0.001**
	No	56 (14.7%)	59 (15.4%)	115 (29.9%)	
Do you chew qat while smoking shisha?	Yes	30 (7.9%)	21 (5.5%)	27 (7%)	**<0.001**
	No	52 (29.9%)	54 (25.6%)	106 (27.5%)	
Are you careful to use your own shisha?	Yes	35 (20.1%)	21 (10%)	56 (14.5%)	**0.004**
	No	37 (21.3%)	37 (17.5%)	74 (19.2%)	
Can you quit smoking the shisha in the future?	Yes	62 (35.6%)	53 (25.1%)	115 (29.9%)	**0.020**
	No	11 (6.3%)	8 (3.8%)	19 (4.9%)	
If any of your best friends ask you to smoke a shisha, do you smoke it?	Yes	57 (32.8%)	41 (19.4%)	98 (25.5%)	**0.003**
	No	112 (64.4%)	163 (77.3%)	275 (71.4%)	

The majority of participants know that WPS causes damage to health (94.8%). About 70% of the participants know that shisha contains nicotine as a cigarette does. Only 58.7% know that shisha contains tobacco as a cigarette does. Approximately 85% of the participants thought that sharing shisha between people could convey diseases. However, only 40% thought that smoking shisha would be less addictive than cigarettes smoking. Half the participants thought that smoking shisha in different flavors, like apple, makes it less harmful than flavorless (Table 4).

**Table 4:** Predictors of WPS.

Predictors	OR	95 % CI	P-value

Male	**1.99**	**1.30, 3.06**	**0.001**
Female	**0.5**	**0.32, 0.76**	**0.001**
Urban	1.27	0.83, 1.95	0.254
Rural	0.78	0.51, 1.19	0.254
Does the water pipe smoking cause damage on health? Agree	0.49	0.15, 1.55	0.250
Does the water pipe smoking cause damage on health? Disagree	1.66	0.26, 10.33	0.583
Does the shisha contain nicotine as cigarette? Agree	1.08	0.66, 1.76	0.736
Does the shisha contain nicotine as cigarette? Disagree	1.25	0.42, 3.74	0.684
Does the shisha contain tobacco as cigarette? Agree	1.28	0.79, 2.07	0.308
Does the shisha contain tobacco as cigarette? Disagree	1.70	0.80, 3.62	0.167
Does the using of shisha between more than one person can convey diseases for you? Agree	0.84	0.40, 1.74	0.642
Does the using of shisha between more than one person can convey diseases for you? Disagree	0.621	0.17, 2.15	0.453
Do you think smoking shisha makes you look good? Yes	1.44	0.37, 5.56	0.59
Do you think smoking shisha makes you look good? No	0.46	0.16, 1.36	0.163
Do you think smoking shisha is less harmful than cigarettes? Yes	**3.84**	**1.88, 7.83**	**<0.001**
Do you think smoking shisha is less harmful than cigarettes? No	1.16	0.62, 2.15	0.632
Do you think smoking shisha will be less addictive than cigarettes smoking? Yes	**3.80**	**2.0, 7.11**	**<0.001**
Do you think smoking shisha will be less addictive than cigarettes smoking? No	**2.16**	**1.13, 4.12**	**0.020**
Do you think the flirt mechanism that in shisha make it less harmful? Yes	**4.84**	**2.51, 9.34**	**<0.001**
Do you think the flirt mechanism that in shisha make it less harmful? No	1.00	0.62, 1.62	0.977
Do you think there are enough awareness campaigns about shisha? Yes	**5.02**	**2.12, 11.91**	**<0.001**
Do you think there are enough awareness campaigns about shisha? No	1.57	0.76, 3.22	0.214
Do you think smoking shisha in different flavor like apple flavor makes it less harmful than flavorless one? Yes	**2.63**	**1.34, 5.15**	**0.005**
Do you think smoking shisha in different flavor like apple flavor makes it less harmful than flavorless one? No	0.76	0.47, 1.22	0.260
Does one of your parents smoke a shisha? Yes	1.22	0.72, 2.08	0.44
Does anyone smoke shisha in your home other than your parents? Yes	**2.61**	**1.68, 4.05**	**<0.001**
Does any of your close friends smoke shisha? Yes	**6.85**	**3.84, 12.22**	**<0.001**
If any of your best friends ask you to smoke a shisha, do you smoke it? Yes	**22.12**	**12.12, 40.37**	**<0.001**

### 3.4. Predictors of WPS

Binary logistic regression analysis demonstrated that males were more likely to smoke shisha than females (Odd Ratio, OR = 1.99, 95% CI [1.30, 3.06], p = 0.001). Interestingly, students who thought that shisha was less harmful and less addictive than cigarettes were more likely to smoke shisha (OR = 3.84, 95% CI [1.88, 7.83], p < 0.001 and 3.80, 95% CI [2.0, 7.11], p < 0.001, respectively). Students who thought that the filter mechanism in shisha made it less harmful were 5 times more likely to smoke shisha (OR 4.84, 95% CI [2.51, 9.34], p < 0.001). Similarly, students who thought that smoking shisha in different flavours, like apple, made it less harmful than flavourless were more likely to smoke shisha (OR = 2.63, 95% CI [1.34, 5.15], p = 0.005). Our analysis showed that parents’ smoking was not a predictor for smoking shisha (OR = 1.22, 95% CI [0.72, 2.08], p = 0.44). However, we found that students who have a smoker close friend had higher odds to smoke shisha than those who had anyone other than parents smoking in their home (OR = 6.85, 95% CI [3.84, 12.22], p < 0.001 and OR = 2.61, 95% CI [1.68, 4.05], p < 0.001, respectively). Therefore, smokers were 22 times more likely to accept their friends’ invitations to smoke than those who had never smoked shisha (OR = 22.12, 95% CI [12.12, 40.37], p < 0.001) (Table 4).

## 4. Discussion

This study aimed to assess the prevalence and predictors of WPS among male and female students at Jazan University. Our findings demonstrated a high prevalence of WPS among university students (34%). The main predictors of WPS were: Being male, the belief that shisha smoking is less harmful & addictive than cigarettes, the belief that the presence of the filter mechanism can protect them, and having a roommate or close friend who smokes. These findings highlight the importance of awareness campaigns to correct misconceptions. In addition, these findings clearly present the significant impact of close friends and roommates on behavior. In the case of cigarette smoking, the influence of the parents in paramount. With WPS, on the other hand, the influence of close friends and roommates is the main factor after the desire and curiosity to try shisha. This may be because shisha is often used in gatherings of friends where it is exchanged between them.

In term of knowledge and attitude, our findings showed that the knowledge of the harm created by WPS was higher in females compared to males (p = 0.032), while males were more likely to be aware with the contents of the shisha (p = 0.003). Females preferred smoking at home and closed places more than males (p = 0.001). This can be explained by the local oriental culture, which prevents girls from smoking in public places. For males, the main causes that made them smoke shisha for the first time were friends and family, while in females, it was the experience and curiosity. Moreover, males were more likely to quit smoking than females (p = 0.020).

Our findings were supported with the previous findings of other studies conducted in Riyadh, Kingdom of Saudi Arabia in 2011 [2]. In one of these previous studies, the prevalence of WPS was high among male and female high school students, which showed that 414 (33%) of the 1272 participants had tried WPS. The current study shows that the result of prevalence was higher than that of the study conducted by Mahfouz et al. in Jazan University (2014), which showed that 14.6% of both male and female students used WPS [12].

In terms of the predictors of WPS, Bashirian et al. demonstrated that the belief in an individual’s ability, support from friends, and the benefits of reducing waterpipe smoking are the most important factors in reducing WPS among students [18]. This supports our observation that friends have a significant impact in whether one will start or quit smoking. Another study by Ziaei and his colleagues reported a significant association between WPS usage and the initial offer of WPS by a close friend (OR = 3.31; 95% CI [2.17, 5.04]) [19]. Moreover, they found that females were associated with lower risk of being WP smokers (OR = 0.45; 95% CI [0.30, 0.70]). These findings were in agreement with ours. Saravanan et al., showed that social interaction is one of the major independent predictors of shisha smoking in comparison to parental smoking behavior. They explained that, due to the religious and legitimate restriction of other forms of substance abuse, students tended towards shisha smoking when they were involved in social interactions in cafeterias and other places [20].

Regarding the effect of family and friends behaviors on encouraging students to practise WPS, 46.9% of the students who had a close smoker friend were frequent WPS users, but 53.1% were non-smokers (p = 0.000). In addition, 37.8% of students who have smoking parents were also smokers; nevertheless, 62.2% of them were non-smokers (p = 0.441). A large number of WPS students have family members at home who smoke, apart from their parents, with a significant association (66,48.2%, P = 0.000), in agreement with the 2014 study of Mahfouz et al. in Jazan [12]. This finding was also consistent with that of Hassan et al. in Riyadh, Saudi Arabia, in 2014, with the participation of Al-Ghad International College students [13]. This previous study found that 18 (48.6%, P = 0.003) of the smoking students stated that most of their friends smoked, 27 (38.0%, P = 0.000) stated that some of their friends smoked, and 16 (44.4%, P=0.7) stated that their fathers smoked. This study also agrees with the results of Al Moamary et al. conducted in Riyadh, Saudi Arabia, in 2011 [2]. They found that the prevalence of WPS among students’ friends was 284 (23.15%, P < 0.001), with a strong relationship existing between WPS students and family members at home, other than their parents, who smoked 132 (10.86%, P < 0.0001) and who encouraged others to use WPS; these findings are in agreement with the current study.

Regarding the place of residence, the prevalence of WPS among students who lived in rural areas (72, 36.7%) was higher than that in urban areas (59, 31.2%), with no significant association (P = 0.253). The previous study conducted in Aleppo, Syria, in 2004 found the same frequency of WPS students in both rural and urban areas (43, 16.0% urban; 43, 13.5% rural) [15].

Moreover, the current study shows that nonmedical students smoked (71, 37%) in greater numbers than medical students who smoked (60, 31.1%), with no significant association (p = 0.223). These frequencies agree with those of the study conducted in Aleppo, Syria, in 2004, in which WPS prevalence was higher among students in science-related areas (50, 17.7%) and arts, law and humanities (24, 13.0%) than among those in medical areas (12, 10.2%) [15]. These findings also agree with the study of Mahfouz et al. in Jazan in 2014 [12].

## Limitations

This study has limitations. First, it acquired a sample from university students rather than the whole population of Jazan City. Second, it used a web-based questionnaire which might not be accessible to all students. Thus, students who use the Internet might have more information about the use and harmful effects of WPS than those who do not. Additionally, the study results cannot be generalised to all residents of the Jazan region.

## Conclusion and recommendations

In conclusion, the prevalence of WPS among college students at Jazan University was high. The influence of siblings and close friends was clearly observed and should be considered in future awareness campaigns. The research team strongly recommends further investigation into this problem, considering that WPS substantially affects one’s health. In addition, they recommend further analysis of the mechanism of WPS to one’s health and the clinical diagnosis and management related to health affected by this lifestyle practice.
