# Correction: Prevalence, Predictors, and Characteristics of Waterpipe Smoking Among Jazan University Students in Saudi Arabia: A Cross-Sectional Study

**DOI:** 10.5334/aogh.4482

**Published:** 2024-06-06

**Authors:** Sarah Salih, Samy Shaban, Zainab Athwani, Faizah Alyahyawi, Sana Alharbi, Fatima Ageeli, Arwa Hakami, Atheer Ageeli, Ohoud Jubran, Saleha Sahloli

**Affiliations:** 1 Jazan University, Saudi Arabia; 2 Public health and community medicine department, Faculty of medicine, Al-Azhar University, Egypt

**Keywords:** Jazan University, waterpipe, shisha, prevelance, predictors, smoking

## Abstract

This article details a correction to: Salih S, Shaban S, Athwani Z, et al. Prevalence, Predictors, and Characteristics of Waterpipe Smoking Among Jazan University Students in Saudi Arabia: A Cross-Sectional Study. *Annals of Global Health*. 2020; 86(1): 87. DOI: https://doi.org/10.5334/aogh.2912

## Correction

The authorial metadata of Salih et al. [1] should have included the following institutional affiliation for Samy Shaban:

Assistant professor, Public health and community medicine department, Faculty of medicine, Al-Azhar University

This affiliation has been added to the metadata of this correction.

## References

[1] Salih S, Shaban S, Athwani Z, et al. Prevalence, Predictors, and Characteristics of Waterpipe Smoking Among Jazan University Students in Saudi Arabia: A Cross-Sectional Study. Annals of Global Health. 2020; 86(1): 87. DOI: 10.5334/aogh.2912 32775218 PMC7394196

